# Relationship between oxygenated fatty acid and milk fat concentration during diet-induced milk fat depression in dairy cows

**DOI:** 10.3168/jdsc.2025-0812

**Published:** 2025-09-10

**Authors:** Y.A. Adeniji, C. Matamoros, R.E. Walker, K.J. Harvatine

**Affiliations:** Department of Animal Science, The Pennsylvania State University, University Park, PA 16802

## Abstract

Altered rumen biohydrogenation of UFA is central to the current understanding of diet-induced milk fat depression (MFD) in dairy cows, and associations with numerous *trans* fatty acids (FA) have been characterized. Although some specific CLA isomers have been demonstrated to be antilipogenic, they fail to account for the full decrease in milk fat synthesis during diet-induced MFD, indicating that other undiscovered bioactive causative actors likely exist. Microbial FA metabolism also results in synthesis of oxy and hydroxy FA and the objective of the current study was to quantify changes in these during diet-induced MFD. We hypothesized that 10-oxo-stearic and 10-hydroxy-stearic acid (10-O-18:0 and 10-OH-18:0) would be increased in milk fat during diet-induced MFD. Milk fat samples from 2 experiments that fed diets resulting in MFD were analyzed. In the first experiment (n = 24), diet-induced MFD increased milk fat concentration of 10-O-18:0 from 0.04% to 0.14% of FA and 10-OH-18:0 from 0.07% to 0.21% of FA, whereas milk fat concentration was decreased 43% and milk fat *trans-*10 18:1 increased from 0.04% to 12.0% of FA. A second experiment that resulted in varying degrees of MFD between the cows was combined to create a dataset for regression analysis (n = 96; *trans-*10 18:1 ranged from 0.27% to 6.69% of FA). In this dataset, 10-O-18:0 and 10-OH-18:0 were quadratically related to *trans-*10 18:1 and ratio of *trans*-10 18:1 to *trans-*11 18:1 in milk fat but not *trans-*11 18:1. These results indicate an association of the oxygenated FA 10-O-18:0 and 10-OH-18:0 with diet-induced MFD. Their causative roles in the physiology of MFD warrant further investigation.

## Highlights

• Oxygenated FA were detected in milk fat by gas chromatography-mass spectrometry (GC-MS).
• Specific oxygenated FA were increased during diet-induced milk fat depression.
• Some oxygenated FA were positively correlated with *trans*-10 18:1.
• Future work is needed to establish possible functional roles of the oxygenated FA.

## Graphical Abstract

**Figure:**
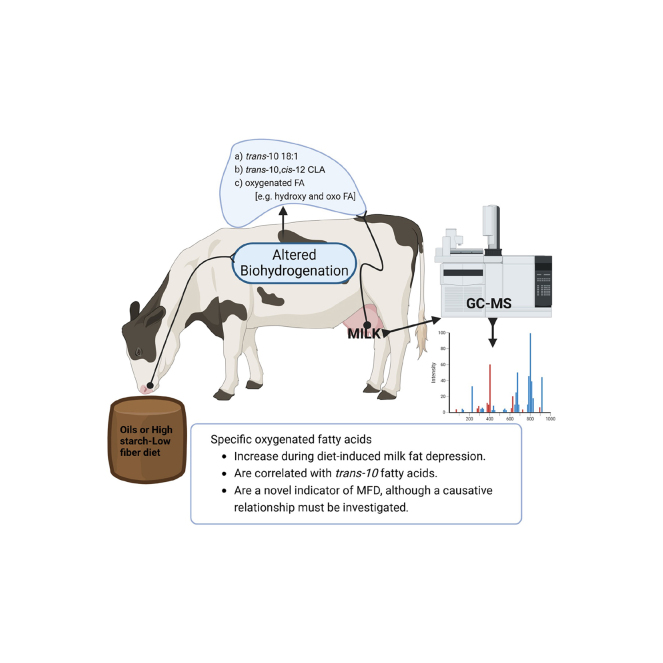
**Summary:** Diet-induced milk fat depression is associated with specific *trans* fatty acids (FA) intermediates of ruminal biohydrogenation of unsaturated FA. Ruminal metabolism also results in the production of oxy and hydroxy FA. These have not been as well investigated, but we characterized their concentration in milk fat during milk fat depression. Both 10-oxostearic acid and 10-hydroxystearic acid were detectable in milk fat, were increased during diet-induced milk fat depression, and were positively correlated with *trans*-10 18:1 previously associated with altered biohydrogenation. These oxygenated FA may be used as markers for milk fat depression, and future work should explore whether they have a causative role in the condition. Graphical abstract created in BioRender (https://BioRender.com/s88w866).

Unsaturated fatty acids (**FA**) comprise a major portion of the FA intake of dairy cows, and they are extensively biohydrogenated in the rumen, resulting in the synthesis of *trans* 18:1 isomers and SFA. Many diverse intermediates are produced in the process, and some have been demonstrated to be bioactive and causative to biohydrogenation-induced milk fat depression (**MFD**; Bauman and Griinari, 2001). Milk fat depression in dairy cows is characterized by up to a 50% reduction in milk fat yield when feeding high concentrate, low-forage diets, or diets supplemented with UFA (Bauman and Griinari, 2003). This limits fat production and results in economic losses to dairy producers. Experiments have extensively investigated dietary and ruminal factors associated with shifting microbial biohydrogenation (**BH**) toward the production of bioactive compounds that directly inhibit mammary milk fat synthesis, for example, *trans-*10,*cis-*12 CLA (Bauman and Griinari, 2001; Jenkins and Harvatine, 2014). However, the magnitude of the increase in *trans-*10,*cis-*12 CLA in milk fat during diet-induced MFD can only explain a limited portion of the decrease in milk fat observed at various degrees of severity of MFD, indicating that other bioactive compounds exist (Bauman and Griinari, 2003). Milk fat *trans-*10 18:1 correlates well with the decrease in milk fat and is commonly used as a biomarker for MFD in the cow due to its higher concentration aiding quantification (Matamoros et al., 2020), but abomasal infusion of pure *trans-*10 18:1 to mid-lactating dairy cows did not reduce milk fat, thus failing to demonstrate a causative role in the condition (Lock et al., 2007). Microbial oxygenation of UFA has also been reported in ovine studies and provides new candidates to investigate during BH-induced MFD (Toral et al., 2024).

Ruminal biohydrogenation of UFA has been well investigated over the past 2 decades (Dewanckele et al., 2020). The traditional pathway of BH of 18:2 n-6 includes isomerization to a CLA isomer, notably *trans*-10,*cis*-12 CLA, before further hydrogenation to *trans*-10 18:1 and finally, 18:0 if the process continues to completion. Metabolism of *cis-*9 18:1 has also been investigated with continuous cultures, although it is more controversial and appears to include both indirect isomerization and direct hydrogenation pathways (Jenkins et al., 2006).

The formation of oxygenated FA in the rumen as intermediates of the hydration of UFA to 10-hydroxystearic acid (**10-OH-18:0**) that is further oxidized to 10-oxostearic acid (**10-O-18:0**) is well described (Jenkins et al., 2006). Previous experiments have observed the conversion of *cis-*9 18:1 to 10-OH-18:0 by bacteria in the rumen digesta of sheep (Kemp et al., 1975) and by nonruminal *Pseudomonas* strains (Wallen et al., 1962), and it was not further metabolized, indicating they are not simply intermediary compounds of the biohydrogenation pathway. These oxygenated FA are products of the hydration of the double bonds in monoenes, and early reports demonstrated synthesis by *Pseudomonas* spp. (Davis et al., 1969). They have also been found in continuous culture fermenters inoculated with rumen fluid obtained from dairy cows and fed canola oil or soybean oil (Jenkins et al., 2006), as well as in vivo in the rumen of sheep fed fish oil (Kitessa et al., 2001; Frutos et al., 2018). The importance of the hydration pathways has not been well explored but provides another option in addition to hydrogenation for rumen microbes to metabolize UFA.

Although changes in oxygenated FA during disrupted rumen fermentation likely originate from rumen metabolism, postabsorptive synthesis of hydroxy FA by oxidizing saturated FA in phospholipids by peroxireoxin-6 has been demonstrated in rats (Benlebna et al., 2021). The regulation of this pathway is not well understood.

Most of the reports of oxygenated FA have been in sheep and intra- and interspecies variation in MFD have been described (Frutos et al., 2018; Della Badia et al., 2021) making it important to examine their relationships with MFD in dairy cows. The objective of the current study was to determine if 10-O-18:0 and 10-OH-18:0 were increased in milk fat during MFD caused by increasing dietary starch and 18:2 n-6 in lactating Holstein dairy cows and to characterize the relationship between these oxygenated FA and other *trans* FA. We hypothesized that both 10-O-18:0 and 10-OH-18:0 would be inversely related to milk fat concentration and positively correlated with *trans-*10 18:1, currently the most well-established biomarkers of BH-induced MFD.

Milk samples from 2 experiments (unpublished; experiment 1 [**Exp. 1**]: C. Matamoros, A. Patterson, and K. J. Harvatine; experiment 2 [**Exp. 2**]: Y. A. Adeniji, R. Bomberger, and K. J. Harvatine; Penn State University, University Park, PA) that fed diets that caused BH-induced MFD in Holstein cows were used for this study. All treatments and animal procedures were approved by the Pennsylvania State University Institutional Animal Care and Use Committee (PROTO202202210 and PROTO202102054 for the first and second experiments, respectively).

Experiment 1 was a 3 × 3 Latin square design with 21-d periods and 12 lactating Holstein cows (6 primiparous with milk yield of 33 ± 5 kg/d and 73 ± 14 DIM, and 6 multiparous with milk yield of 53 ± 7 kg/d and 94 ± 28 DIM at the start of the experiment; only samples from the control and BH-induced MFD treatment were analyzed [n = 24]). The higher fiber and lower UFA control diet contained 32.8% NDF, 26.5% starch, and no added soybean oil, whereas the MFD diet decreased NDF to 28.4%, increased starch to 31.8%, and included 2.1% soybean oil. Cows were milked at ∼0700 and 1800 h. Two milk samples were collected on d 20. One was preserved with bronopol (Bronolab-WII; D&F Control Systems Inc., Dublin, CA), and the milk fat concentration determined by infrared spectroscopy (DairyOne DHIA, Ithaca NY). The second sample was composited based on milk yield and fat extracted using hexane-isopropanol and FA transmethylated with sodium methoxide (Rico and Harvatine, 2013). The FA profile was first analyzed by GC with a capillary column (100 m × 0.25 mm i.d. with a 0.2-µm film thickness; SP-2560, Supelco Inc., Bellefonte, PA) and a flame ionization detector, as described by Baldin et al. (2018). Oxygenated FA were then quantified by a GC coupled to a quadrupole mass selective detector (5973N, Agilent Technologies Inc.) and a capillary column (30 m × 0.25 mm i.d. with 0.25-µm film thickness; DB-FastFAME, Agilent Technologies, Inc.) in selected ion monitoring mode. The filament trap current was 400 μA at 70 eV and the minimum and maximum mass set to 30 and 500 amu, respectively. The carrier gas was helium at a constant flow of 1 mL/min. Samples (1 µL) were injected with a 15:1 split. The inlet and detector temperature were 250°C, and the oven was initially 140°C for 3 min, then increased 2°C/min until a final temperature of 220°C, and then held for 2 min. The FA spectra were identified using a combination of the NIST 11 (https://chemdata.nist.gov/) and Lipid Maps (https://www.lipidmaps.org/) libraries and a 10-hydroxy-stearic methyl ester standard (Nu-Chek Prep Inc., Elysian, MN).

First, the effect of BH-induced MFD was tested using a mixed model that included the random effects of period and cow and the fixed effect of treatment (JMP Pro 16, SAS Institute Inc.). Both 10-O-18:0 and 10-OH-8:0 required log-transformation, and back-transformed data are reported. Data with Studentized residuals outside of ±3.0 were considered outliers and removed from the analysis. Significance was declared at *P* ≤ 0.05.

The induction of MFD by increasing diet fermentability and 18:2n-6 concentration decreased milk fat concentration from 3.65% to 2.20% and increased *trans*-10 18:1 from 0.61% to 8.16% of FA (data not shown). Both 10-O-18:0 and 10-OH-18:0 were detected in milk fat from CON (0.07% and 0.04% of FA). The highly fermentable and high-PUFA diet that caused MFD resulted in a 2-fold increase in 10-O-18:0 and 2.5-fold increase in 10-OH-18:0 (0.15% and 0.22% of FA, respectively; Figure 1). These FA have also been reported in the rumen (Kairenius et al., 2011) and milk fat of ewes (Frutos et al., 2018) and cows (Kairenius et al., 2015) that were fed diets containing marine lipids and that experienced some degree of MFD indicated by increased *trans*-10 18:1.

**Figure 1:**
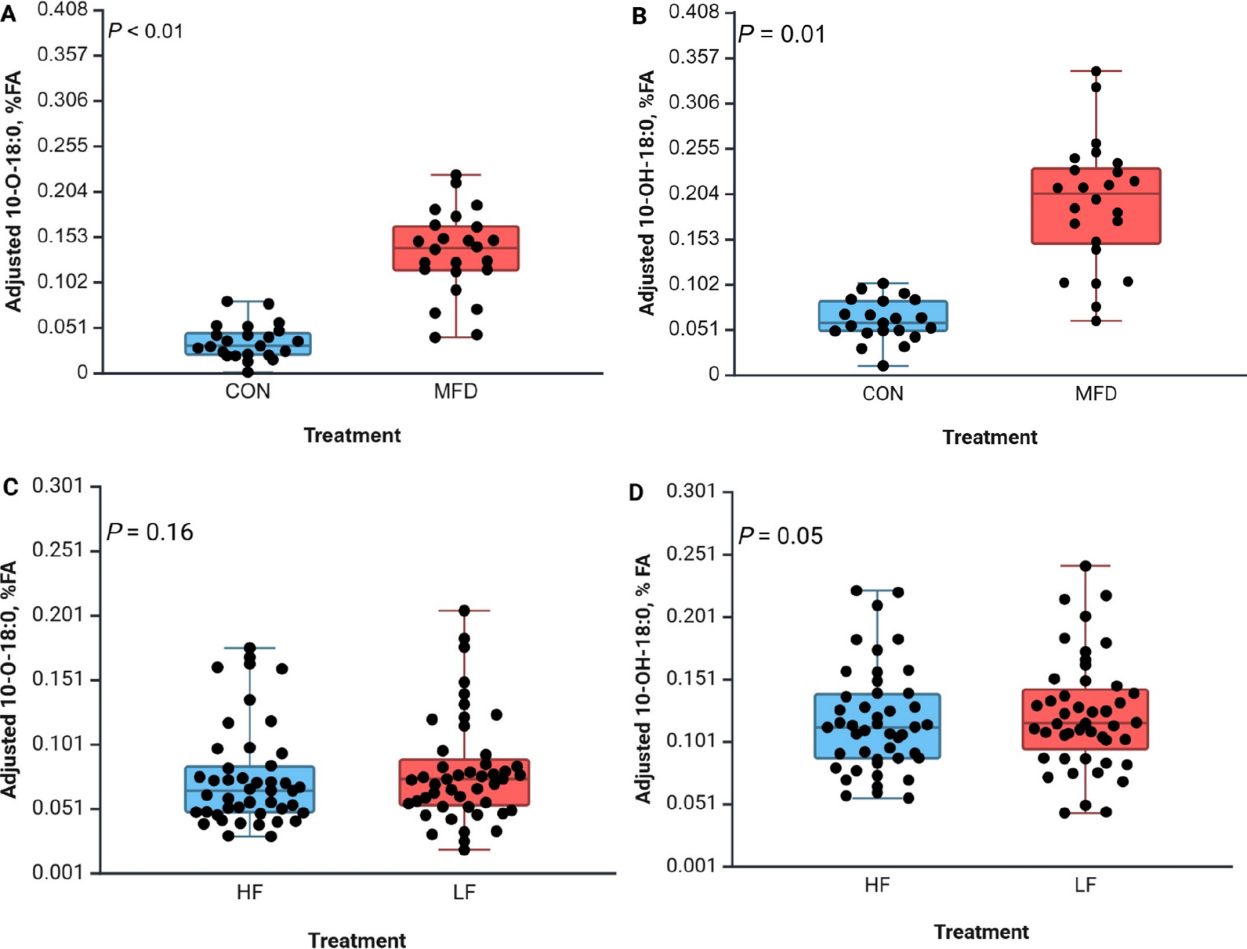
Effect of diet-induced MFD on the concentration of 10-O-18:0 (A, C) and 10-OH-18:0 (B, D) in milk fat of cows fed a higher fiber and lower UFA diet (CON) or a diet higher in starch and UFA that resulted in a 43% decrease in milk fat yield (MFD) in Exp. 1 and effect of a higher fermentable basal diet (LF) that increased dietary fat with 2% blend of palmitic and stearic acid (HF), respectively, in Exp. 2. Plotted data points were adjusted for the random effects of cow and period. Box plots show the mean (midline), upper and lower quartiles (upper and lower edges of boxes), and SEM bars, and the effect of treatment is shown in each panel.

Samples from Exp. 2 (Adeniji et al., 2023) with 48 cows (20 primiparous with milk yield of 49.2 ± 11.2 kg/d and 116 ± 10 DIM, and 28 primiparous with milk yield of 39.9 ± 6.43 kg/d and 189 ± 24 DIM at the start of the experiment) that experienced varying degrees of MFD were used to create a larger combined database for regression analysis. The second experiment was a crossover design (28-d period and 7-d washout) and investigated the interaction of pretrial milk fat production level on response to increasing dietary fat. Treatments were a low-fat diet balanced to 24.9% NDF, 29.2% starch, and 3.49% total FA on a DM basis, and a diet that contained an FA supplement that was a mixture of palmitic and stearic acid at 2% of diet DM. Milk was sampled and FA profile quantified using a process similar to that described for Exp. 1. Although the basal diet was not balanced intending to cause BH-MFD, average milk fat concentration was lower than expected (3.49%; ranged from 1.60% to 5.64%) and the concentration of *trans-*10 18:1 in milk fat was also higher than expected (2.32% of FA; range 0.27% to 6.69% FA), indicating that cows experienced varying degrees of BH-induced MFD (description of milk and milk fatty acid profile available in Supplemental Table S1: see Notes). This was likely due to a high feeding rate of corn silage, low feeding rate of haylage, higher than expected diet fermentability, and heat stress, as the experiment was conducted during the summer.

Random regression analysis was conducted using the combined dataset from Exp. 1 and 2, and the model included the random effect of experiment and period nested in experiment (JMP Pro 16; SAS Institute Inc.). Plotted data were adjusted for the random effects.

We observed a linear relationship between both 10-O-18:0 and 10-OH-18:0 and milk fat concentration (partial R^2^ = 0.41 and 0.44 and root mean square error [**RMSE**] = 0.44 and 0.54; *P* < 0.001; Figure 2). Cows experiencing mild to no MFD (milk fat ≥3.0%) had 10-OH-18:0 and 10-O-18:0 values below 0.10% and 0.05% of FA, respectively. We found a clear decline in milk fat with increasing concentrations of 10-OH-18:0 and 10-O-18:0 in milk fat and reached a nadir at 2% milk fat with the oxygenated FA at ∼0.1% and 0.15% of FA, respectively.

**Figure 2:**
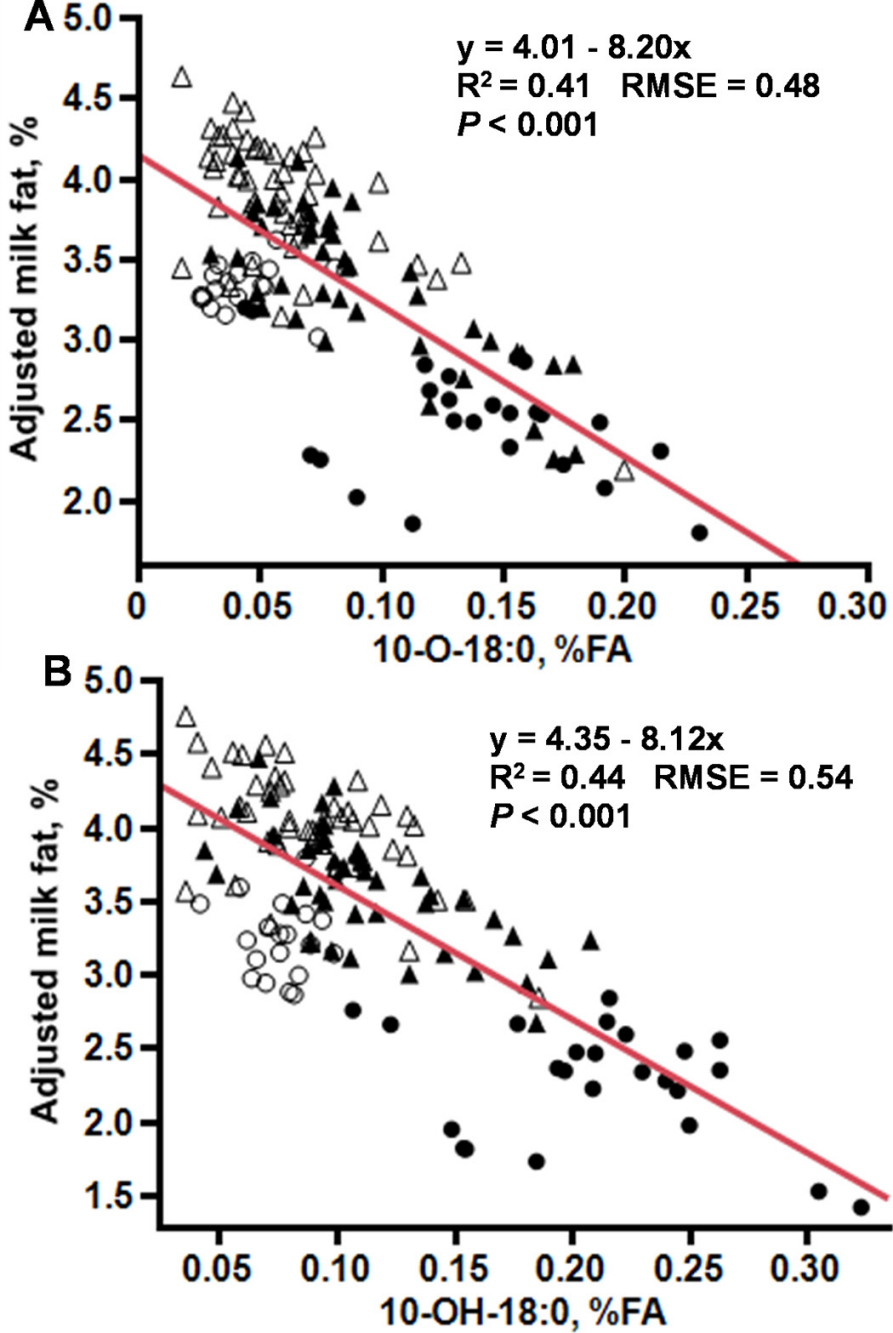
The relationship between milk fat concentration and 10-OH-18:0 and 10-O-18:0 in milk fat of cows with varying level of milk fat depression. Data are from 2 experiments. In Exp. 1 (n = 24; 12 cows), cows were fed either a lower fermentability UFA diet (○) or a higher fermentability UFA diet (•). In Exp. 2 (n = 96; 48 cows, crossover design), cows experienced varying levels of milk fat depression when fed either a lower-fat basal diet (Δ) or the same basal diet supplemented with 2% palmitic and stearic acid blend (▴). Data points shown are adjusted for the random effects of cow, period, and experiment. Partial R^2^ of the fit line is shown.

The relationship between *trans*-10 18:1 in milk fat and 10-OH-18:0 and 10-O-18:0 was also explored using the combined dataset (n = 144) and with Exp. 2 only (n = 96). Across the 2 experiments, we observed a quadratic relationship between 10-OH-18:0 and *trans-*10 18:1 (partial R^2^ of 0.48 and RMSE of 0.03) and a linear relationship between and 10-O-18:0 and *trans-*10 18:1 (partial R^2^ of 0.50 and RMSE of 0.03; Figure 3). This demonstrates a positive relationship with an established biomarker of BH-induced MFD. It is important to note that nearly all of the *trans*-10 18:1 points above 7% of FA were from the diet-induced MFD treatment in Exp. 1. Thus, regression analysis was also conducted with only Exp. 2 data. The quadratic relationship between 10-OH-18:0 and 10-O-18:0 and *trans-*10 18:1 remained (partial R^2^ = 0.48 and 0.34, respectively; *P* < 0.001).

**Figure 3:**
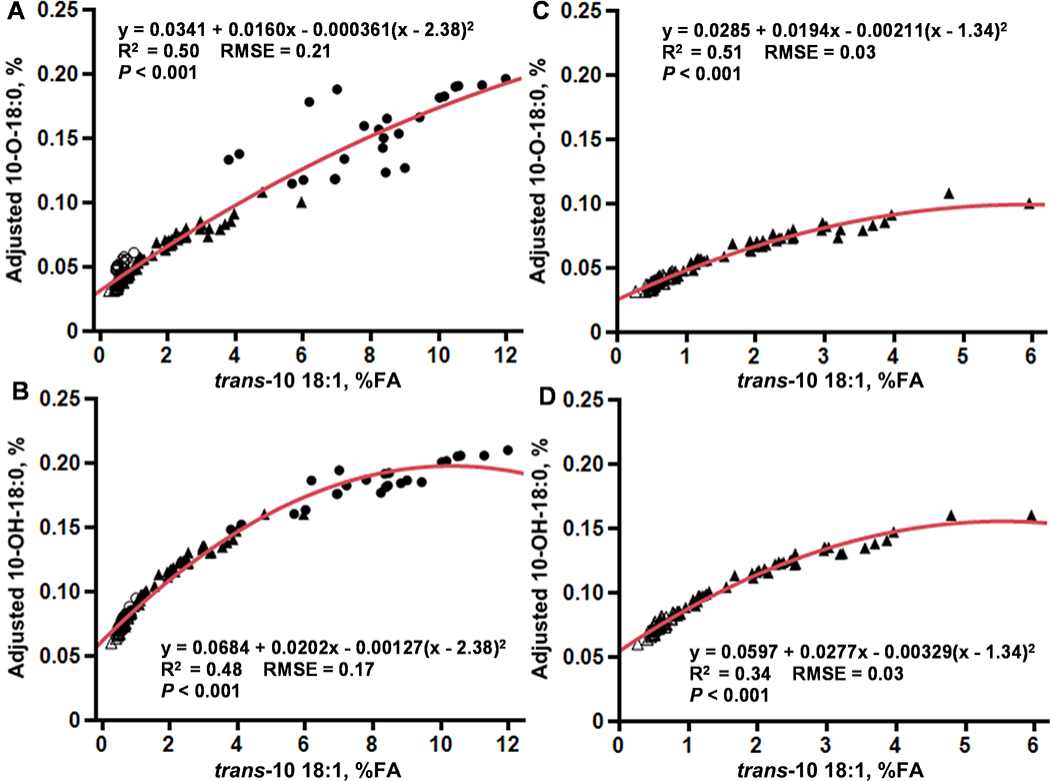
Relationship between *trans*-10 18:1 and 10-OH-18:0 and 10-O-18:0 in milk fat of cows with varying levels of milk fat depression. Data were used from 2 experiments. In Exp. 1 (n = 24; 12 cows), cows were fed either a lower fermentability UFA diet (○) or a higher fermentability UFA diet (•). In Exp. 2 (n = 96; 48 cows, crossover design), cows experienced varying levels of milk fat depression when fed either a lower-fat basal diet (Δ) or the same basal diet supplemented with 2% palmitic and stearic acid blend (▴). In panels A and B, data from both experiments was analyzed together using a random regression model and data from Exp. 2 was analyzed alone in panels C and D. Plotted data points are adjusted for the random effects of cow and period. Partial R^2^ of the fit line is shown.

Oxygenated FA have been reported to be products of ruminal hydration of oleic acid in vitro (Jenkins et al., 2006), and bacterial hydration of linoleic acid to 10-OH-18:0 has been reported (Takatori, 2001). In vivo, it is more difficult to discern the substrate for their synthesis. However, it is possible that these oxygenated FA are simply produced alongside *trans-*10 18:1 and other bioactive in the rumen as the conversion of *trans-*10 18:1 to either 10-O-18:0 or 10-OH-18:0 has not been reported. The oxygenated FA increased the shift to the alternate biohydrogenation pathway, calculated as the ratio of *trans*-10 18:1 to *trans*-11 18:1 (partial R^2^ of 0.44), but there was no relationship with *trans*-11 18:1 alone (partial R^2^ of 0.01; data not shown). This shift in biohydrogenation intermediates has been associated with diet-induced MFD in dairy cows, with CLA believed to be the major cause of reduced milk fat synthesis due to its potent antilipogenic properties (Baumgard et al., 2001). However, there we did not observe similar relationship with CLA and is important to state that we are limited in our analysis because CLA was not detectable in more than half of our dataset. This relationship has not been previously reported, and these correlations do not demonstrate a causative role of these oxygenated compounds. Additional work is necessary to specifically test their direct effects on milk fat synthesis.

Previously, Márquez-Ruiz et al. (2011) observed an increase in *trans*-10 18:1 and 10-OH-18:0 and 10-ketostearic acids in milk fat when dietary *cis-*9 18:1 was increased in ewes and goats. In their experiment, only trace amount of 10-OH-18:0 (0.08% of FA) were found in milk fat when fed the control diet, but when ewes were fed diets supplemented with a mixture of 2.5% sunflower oil plus 0.8% or 1.6% algae oil, the concentration of 10-OH 18:0 in milk fat increased over 4 fold, whereas the concentration of *trans*-10 18:1 increased from 0.4% to ∼10% of FA. Other studies have also observed increased concentrations of 10-O-18:0 in sheep fed diets containing fish oil (e.g., Frutos et al., 2018; Toral et al., 2018).

In the current dataset, the relationship between the oxygenated FA with milk fat and other FA, including 18:2n-6, <16 C, 16 C, >16 C, the sum of odd- and branched-chain FA (**OBCFA**), and the sum of *trans*-4 18:1 to *trans*-12 18:1 (total *trans*), was also examined by principal component analysis (supplemental data; see Notes) and resulted in 2 distinct clusters with the first and second principal components explaining 66% of the variation. Milk fat concentration and milk fat <16 C FA, 16 C FA, and OBCFA as a percent of milk FA were positively correlated and clustered together, whereas 10-O-18:0, 10-OH-18:0, 18:2 n-6, >16 C FA, *trans-*10 18:1, and total *trans* clustered together and were negatively correlated with milk fat concentration (r = −0.59, −0.60, −0.40, −0.60, −0.78, −0.65, respectively).

Recently fatty acid esters of hydroxy fatty acids (**FAHFA**) have been identified as a family of bioactive lipids and been implicated in insulin resistance and possibly other metabolic disorders (Moraes-Vieira et al., 2016). The ability of FAHFA to inhibit milk fat synthesis has not been explored, but the increases in 10-O-18:0 and 10-OH-18:0 may simply correlate with changes in FAHFA or other bioactives derived from the oxygenated FA.

In conclusion, the oxygenated FA 10-O-18:0 and 10-OH-18:0 were quantifiable in milk fat of dairy cows and were both increased during diet-induced MFD. Additionally, their concentration in milk FA was inversely related to milk fat concentration and positively correlated with *trans-10* 18:1, which is the most well-established biomarker of BH-induced MFD. Their pathways of ruminal synthesis and functional role as bioactives causing MFD warrants further investigation.

## References

[bib1] Adeniji Y., Bomberger R., Harvatine K. (2023). Interaction of pretrial milk fat production and dietary fat supplementation on milk and milk fat yield in Holstein cows. J. Dairy Sci..

[bib2] Baldin M., Zanton G.I., Harvatine K.J. (2018). Effect of 2-hydroxy-4-(methylthio)butanoate (HMTBa) on risk of biohydrogenation-induced milk fat depression. J. Dairy Sci..

[bib3] Bauman D., Griinari J. (2001). Regulation and nutritional manipulation of milk fat: Low-fat milk syndrome. Livest. Prod. Sci..

[bib4] Bauman D.E., Griinari J.M.M. (2003). Nutritional regulation of milk fat synthesis. Annu. Rev. Nutr..

[bib5] Baumgard L.H., Sangster J.K., Bauman D.E. (2001). Milk fat synthesis in dairy cows is progressively reduced by increasing supplemental amounts of *trans*-10, *cis*-12 conjugated linoleic acid (CLA). J. Nutr..

[bib6] Benlebna M., Balas L., Gaillet S., Durand T., Coudray C., Casas F., Feillet-Coudray C. (2021). Potential physio-pathological effects of branched fatty acid esters of hydroxy fatty acids. Biochimie.

[bib7] Davis E.N., Wallen L.L., Goodwin J.C., Rohwedder W.K., Rhodes R.A. (1969). Microbial hydration of *cis*-9-alkenoic acids. Lipids.

[bib8] Della Badia A., Hervás G., Toral P.G., Frutos P. (2021). Individual differences in responsiveness to diet-induced milk fat depression in dairy sheep and goats. J. Dairy Sci..

[bib9] Dewanckele L., Toral P.G., Vlaeminck B., Fievez V. (2020). Invited review: Role of rumen biohydrogenation intermediates and rumen microbes in diet-induced milk fat depression: An update. J. Dairy Sci..

[bib10] Frutos P., Toral P.G., Belenguer A., Hervás G. (2018). Milk fat depression in dairy ewes fed fish oil: Might differences in rumen biohydrogenation, fermentation, or bacterial community explain the individual variation?. J. Dairy Sci..

[bib11] Jenkins T.C., Abughazaleh A.A., Freeman S., Thies E.J. (2006). The production of 10-hydroxystearic and 10-ketostearic acids is an alternative route of oleic acid transformation by the ruminal microbiota in cattle. J. Nutr..

[bib12] Jenkins T.C., Harvatine K.J. (2014). Lipid feeding and milk fat depression. Vet. Clin. North Am. Food Anim. Pract..

[bib13] Kairenius P., Ärölä A., Leskinen H., Toivonen V., Ahvenjärvi S., Vanhatalo A., Huhtanen P., Hurme T., Griinari J.M., Shingfield K.J. (2015). Dietary fish oil supplements depress milk fat yield and alter milk fatty acid composition in lactating cows fed grass silage-based diets. J. Dairy Sci..

[bib14] Kairenius P., Toivonen V., Shingfield K.J. (2011). Identification and ruminal outflow of long-chain fatty acid biohydrogenation intermediates in cows fed diets containing fish oil. Lipids.

[bib15] Kemp P., White R.W., Lander D.J. (1975). The hydrogenation of unsaturated fatty acids by five bacterial isolates from the sheep rumen, including a new species. J. Gen. Microbiol..

[bib16] Kitessa S., Gulati S., Ashes J., Fleck E., Scott T., Nichols P. (2001). Utilisation of fish oil in ruminants. Anim. Feed Sci. Technol..

[bib17] Lock A.L., Tyburczy C., Dwyer D.A., Harvatine K.J., Destaillats F., Mouloungui Z., Candy L., Bauman D.E. (2007). *Trans*-10 octadecenoic acid does not reduce milk fat synthesis in dairy cows. J. Nutr..

[bib18] Márquez-Ruiz G., Rodríguez-Pino V., de la Fuente M.A. (2011). Determination of 10-hydroxystearic, 10-ketostearic, 8-hydroxypalmitic, and 8-ketopalmitic acids in milk fat by solid-phase extraction plus gas chromatography-mass spectrometry. J. Dairy Sci..

[bib19] Matamoros C., Klopp R.N., Moraes L.E., Harvatine K.J. (2020). Meta-analysis of the relationship between milk *trans*-10 C18:1, milk fatty acids <16 C, and milk fat production. J. Dairy Sci..

[bib20] Moraes-Vieira P.M., Saghatelian A., Kahn B.B. (2016). GLUT4 expression in adipocytes regulates de novo lipogenesis and levels of a novel class of lipids with antidiabetic and anti-inflammatory effects. Diabetes.

[bib21] Rico D.E., Harvatine K.J. (2013). Induction of and recovery from milk fat depression occurs progressively in dairy cows switched between diets that differ in fiber and oil concentration. J. Dairy Sci..

[bib22] Takatori T. (2001). The mechanism of human adipocere formation. Leg. Med. (Tokyo).

[bib23] Toral P.G., Hervás G., Peiró V., Frutos P. (2018). Conditions associated with marine lipid-induced milk fat depression in sheep cause shifts in the in vitro ruminal metabolism of 1-^13^C oleic acid. Animals (Basel).

[bib24] Toral P.G., Hervás G., Frutos P. (2024). Invited review: Research on ruminal biohydrogenation—Achievements, gaps in knowledge, and future approaches from the perspective of dairy science. J. Dairy Sci..

[bib25] Wallen L.L., Benedict R.G., Jackson R.W. (1962). The microbiological production of 10-hydroxystearic acid from oleic acid. Arch. Biochem. Biophys..

